# Rapid Plasma-Assisted Synthesis of Tannic Acid-Capped Gold Nanoparticles for SERS Applications

**DOI:** 10.3390/nano16211386

**Published:** 2026-10-28

**Authors:** Nguyen Thi Huyen, Nguyen Quynh Chi, Nguyen Thu Loan, Simon Chouteau, Jarl Tynan Collado, Pierre Vinchon, Vu Ngoc Linh, Man Minh Tan, Nguyen Hoang Tung, Nguyen Thanh Tung, Nguyen Nhat Linh

**Affiliations:** 1 Laboratory of Advanced Materials and Natural Resources, Institute for Advanced Study in Technology, Ton Duc Thang University, Ho Chi Minh City 758307, Vietnam; nguyenthihuyen1@tdtu.edu.vn (N.T.H.); manminhtan@tdtu.edu.vn (M.M.T.); 2 Faculty of Applied Sciences, Ton Duc Thang University, Ho Chi Minh City 758307, Vietnam; 3 Graduate University of Science and Technology, Vietnam Academy of Science and Technology, 18 Hoang Quoc Viet, Nghia Do, Hanoi 10072, Vietnam; 4 Institute of Materials Science, Vietnam Academy of Science and Technology, 18 Hoang Quoc Viet, Nghia Do, Hanoi 10072, Vietnam; chinq@ims.vast.ac.vn (N.Q.C.); loannt@ims.vast.ac.vn (N.T.L.); tungnh@ims.vast.ac.vn (N.H.T.); tungnt@ims.vast.ac.vn (N.T.T.); 5 R^3^ Institute of Newly-Emerging Science Design (INSD), The University of Osaka, Osaka 565-0871, Japan; chouteau.simon.insd@osaka-u.ac.jp (S.C.); jarl.collado.8qs@ecs.osaka-u.ac.jp (J.T.C.); vinchon.pierre.insd@osaka-u.ac.jp (P.V.); 6 University of Engineering and Technology, Vietnam National University, VNU Hoa Lac Campus, Hoa Lac Commune, Hanoi 100000, Vietnam; vungoclinh@vnu.edu.vn; 7 University of Science and Technology of Hanoi, Vietnam Academy of Science and Technology, 18 Hoang Quoc Viet, Nghia Do, Hanoi 10072, Vietnam

**Keywords:** plasma–liquid interaction, tannic acid, gold nanoparticles, green synthesis, X-ray photoelectron spectroscopy, surface-enhanced Raman scattering

## Abstract

Gold nanoparticles (AuNPs) are attractive substrates for surface-enhanced Raman scattering (SERS) because of their strong visible plasmon resonance, chemical stability, and biocompatibility. However, the enhancement they provide depends on how accessible the gold surface is to the analyte. Tannic acid (TA) is an effective green reductant and stabilizer for AuNPs. Conventional TA-based syntheses, however, require large doses and boiling, leaving a dense organic shell on the particles. Herein, an argon cold atmospheric-pressure plasma jet combined with a low dose of TA reduced HAuCl_4_ to AuNPs at room temperature without additional chemical reductants. Well-dispersed colloids of quasi-spherical, crystalline particles about 10 nm in size formed with 25-fold less TA than was required without plasma. In situ UV–Vis spectroscopy showed that low-dose TA largely removed the induction period of plasma reduction and that particle growth continued after the discharge was switched off. This result suggests that plasma supplies most of the reducing power, whereas TA stabilizes the nascent nuclei. XPS confirmed metallic gold, with a small positive Au 4f shift consistent with surface-bound TA. The lightly capped AuNPs gave stronger methylene blue SERS signals than both TA-free plasma AuNPs and AuNPs prepared with 25-fold more TA. This room-temperature route therefore provides clean colloidal AuNPs as promising SERS substrates for chemical sensing.

## 1. Introduction

Surface-enhanced Raman scattering (SERS) is a nanomaterial-based sensing technique that offers simple operation and high sensitivity [1,2,3]. Gold nanoparticles are the most commonly used nanomaterials for SERS because their strong localized surface plasmon resonance (SPR) generates intense electromagnetic “hot spots” at and between particle surfaces [4,5,6]. Because this enhancement decays within a few nanometers of the metal, SERS performance depends not only on particle size and uniformity but critically on how accessible the metal surface is to the analyte. Surface coatings can restrict analyte access to nanoparticles [1]. Stabilizers and synthesis residues adsorbed on the particles compete with the analyte for adsorption sites and hot spots, add their own Raman background, and lower the sensitivity [7,8,9]. In this context, removing or thinning the capping layer strengthens the sensing response. Conventional chemical synthesis based on the Turkevich method, however, relies heavily on sodium citrate as a stabilizer, which affects the sensitivity of the AuNP product. For example, oxidative removal of the citrate coating on AuNPs lowered the detection limit of model analytes tenfold [10]. Post-synthetic remedies, such as repeated washing, ligand exchange, or oxidative removal of the capping agent, can require harsh chemicals that may be harmful to humans and ecosystems [7,10,11]. A more direct strategy is to minimize the stabilizer at the synthesis stage, so that the particles leave the reaction with only the capping layer needed to keep them dispersed.

In recent years, cold atmospheric-pressure plasma has been used to fabricate AuNPs. At the plasma–liquid interface, the discharge injects solvated electrons (e^−^aq), hydrogen radicals (H•), and other reactive species into the liquid boundary layer, where they reduce metal ions at room temperature without added chemical reductants [12,13,14]. Plasma syntheses of AuNPs fall broadly into two groups. Stabilizer-free approaches yield surfactant-free particles, but they tend to be large and polydisperse [15,16,17]. Polymer- or biomolecule-assisted routes give more stable colloids and can increase the amount of gold converted to particles. Still, researchers typically add the macromolecule in amounts comparable to the gold, and it remains on the particle surface [18]. Plasma reduction of Au^3+^ is nevertheless not instantaneous. Radiation-chemistry studies show that although e^−^aq is among the strongest and fastest reductants, the reduction of AuCl_4_^−^ to Au^0^ is a multistep, nonlinear process that passes through Au^+^ intermediates [18]. The result is an induction window during which Au^3+^ is being reduced, but no detectable nuclei and no SPR band appear [19]. In plasma synthesis, this induction window is a few minutes and reflects the discharge energy and the time spent before any particle forms [20,21].

We hypothesize that a low TA dose can address this limitation. TA is a plant-derived polyphenol widely used in the green synthesis of AuNPs, where it acts as both a reductant and stabilizer [22,23,24]. TA exhibits antioxidant properties, allowing it to reduce the AuCl_4_^−^ precursor to an Au^+^ intermediate, which can be further reduced to Au^0^ [23]. Because TA is a mild reductant, TA-based syntheses typically require high TA: Au molar ratios and are usually performed in boiling solution to accelerate reduction [22]. The resulting excess polyphenol forms a thick organic shell on the particles, restricting access to the gold surface and limiting performance in applications such as SERS.

Partial reduction of Au^3+^ to Au^+^ by TA before and during plasma exposure would lower the available Au^3+^ concentration, so that plasma-generated e^−^_aq_ reduce Au^+^ directly to Au^0^, and nucleation would begin sooner [18]. TA may also scavenge plasma-generated •OH [25,26]. Once nuclei form, the same polyphenol caps the particles and restrains growth. TA would then be needed only in the small quantity required to pre-reduce and cap, not in the large excess required to reduce Au^3+^ to Au^0^ on its own.

In this work, an argon cold atmospheric-pressure plasma jet was combined with a low dose of TA to synthesize AuNPs from aqueous HAuCl_4_ at room temperature (AuNPs-TA-Plasma). After characterizing the plasma jet by optical emission spectroscopy, we examine how HAuCl_4_ concentration, TA dose, treatment time, and argon flow rate govern particle yield and uniformity using UV–Vis spectroscopy, and we test the route in a batch fivefold larger. We characterize the optimized particles by SEM, TEM, and HRTEM, and use XPS to compare the surface of gold produced by plasma alone with that produced by plasma and TA. We then use in situ UV–Vis spectroscopy during continuous and intermittent plasma treatment to identify how TA accelerates plasma-driven reduction. Finally, using methylene blue (MB) as the probe, we compare the SERS performance of AuNPs-TA-Plasma with that of AuNPs synthesized by plasma without TA (AuNPs-Plasma) and AuNPs synthesized with a high dose of TA without plasma (AuNPs-TA). By combining plasma-driven reduction with growth control by a green polyphenol at a fraction of its conventional dose, the approach aims to accelerate particle formation and leave a cleaner, more accessible particle surface for SERS.

## 2. Materials and Methods

### 2.1. Chemicals

Gold(III) chloride trihydrate (HAuCl_4_·3H_2_O, ≥99.9%, Sigma-Aldrich, USA) was dissolved in deionized (DI) water to prepare a 10 mM stock solution. Tannic acid (TA, Sigma-Aldrich, USA) was dissolved in DI water at 1.7 mg mL^−1^ (1.0 mM). We used sodium hydroxide (NaOH, China, ≥97%) and hydrochloric acid (HCl, Merck, Germany, 37%) to adjust the pH. Methylene blue (MB, Fuchen, China, ≥98.5%) and Rhodamine B (RhB, Fisher, USA) were used as the SERS analytes.

### 2.2. Plasma Jet

**Figure 1:**
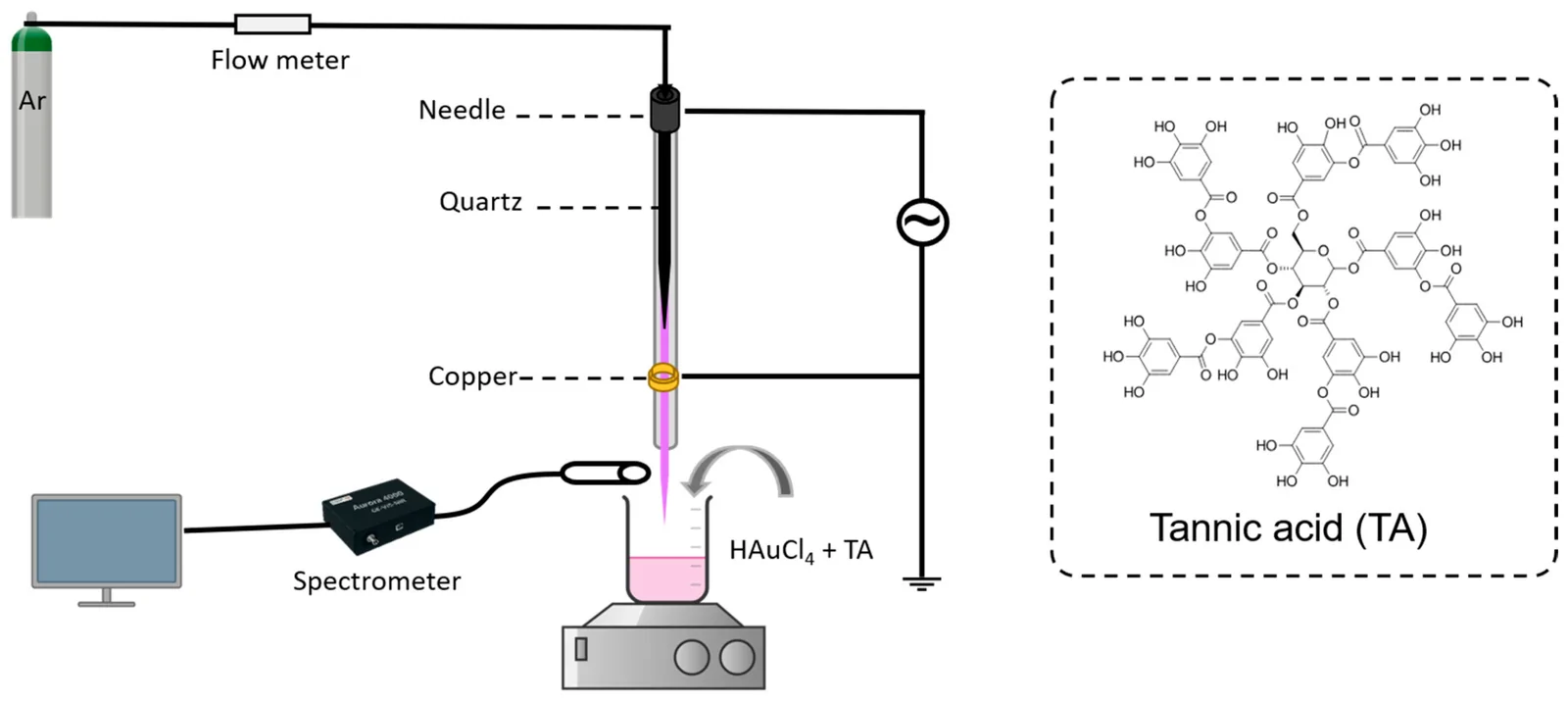
Schematic of the experimental set-up of argon cold atmospheric-pressure plasma jet for the fabrication of AuNPs-TA-Plasma.

We used a custom-built atmospheric-pressure plasma jet (Figure 1). We inserted a stainless-steel syringe needle, serving as the high-voltage electrode, into a quartz capillary tube (10 cm long, 4 mm outer diameter, 2 mm inner diameter) that served as the dielectric barrier. A copper adhesive tape around the capillary’s outer surface served as the grounded electrode. An AC power supply drove the discharge at an applied voltage of 5 kV. Argon was used as the working gas. A mass-flow controller regulated the Ar flow rate from 1 to 2 L min^−1^. Figure 1 shows the plasma jet set-up used in this work.

**Figure 2:**
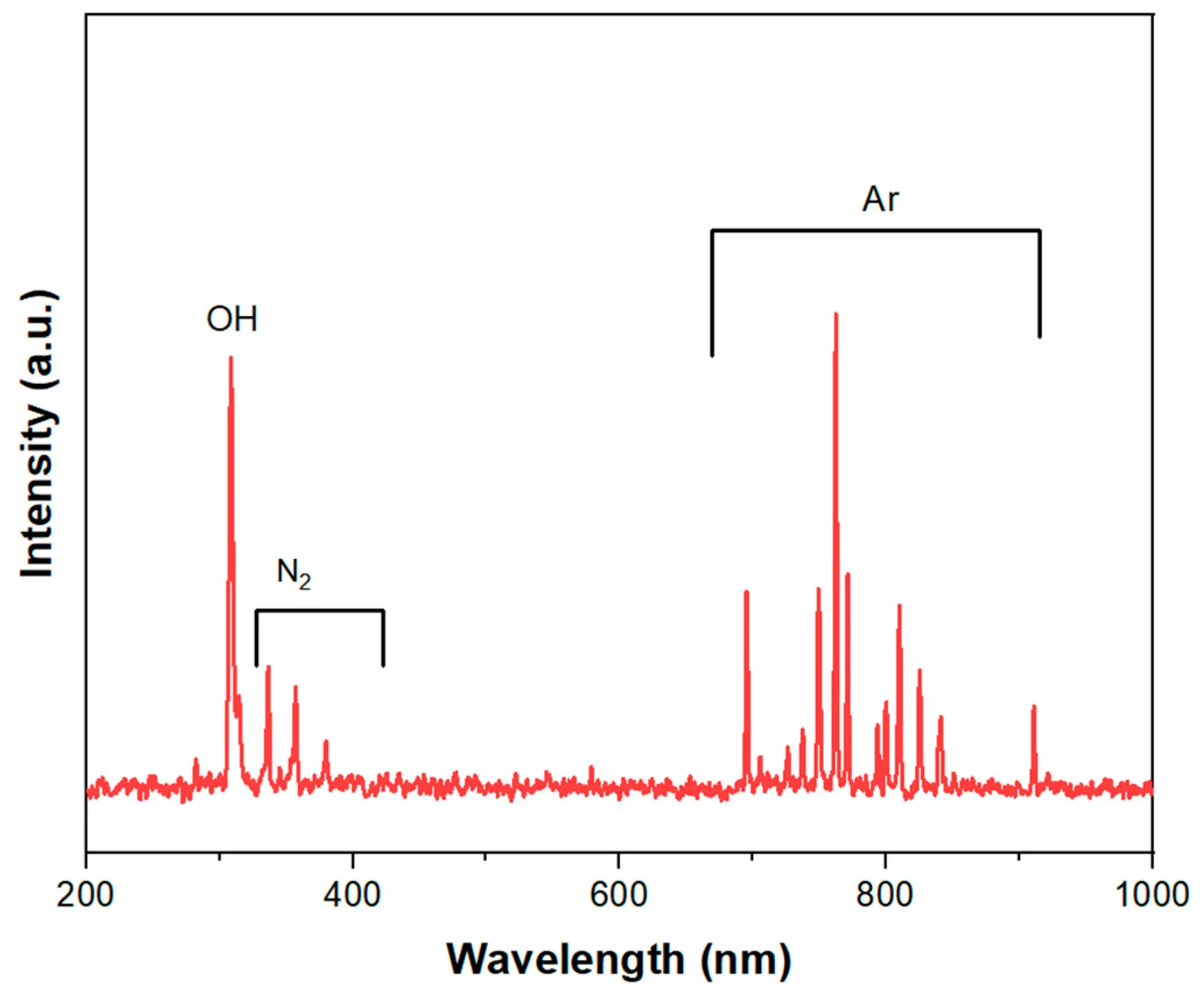
Optical emission spectrum of the plasma jet, showing OH (309 nm), N_2_ SPS (330–380 nm), and Ar I (690–920 nm) emission.

The optical emission spectrum of the plasma plume is demonstrated in Figure 2. A strong emission band of OH(A^2^Σ^+^ → X^2^Π) was observed at 309 nm [27]. OH is formed by dissociative quenching of Ar metastables and by electron-impact dissociation of H_2_O molecules in ambient air [28]. The N_2_ second positive system (SPS C^3^Π_u_ → B^3^Π_g_) at 337, 358, and 380 nm arises from air entrained into the argon plume [29]. Ar I (2p-1s) lines can be observed between 690 and 920 nm [30].

### 2.3. Synthesis of AuNPs

We carried out the reactions in a glass vial containing 10 mL of 0.2 mM HAuCl_4_ precursor solution under magnetic stirring. The distance between the jet nozzle and the liquid surface was fixed at 10 mm. We added an aliquot of 1 mM TA stock (20 µL) to the HAuCl_4_ solution, then treated it with the plasma jet for 10 min. The TA-free plasma controls were prepared under similar conditions without TA. In the plasma-free controls, we added 10–500 µL of TA stock to 0.2 mM HAuCl_4_ at room temperature and kept the reaction time at 10 min. Throughout this paper, we denote the samples as AuNPs-TA-Plasma (plasma with TA), AuNPs-Plasma (plasma without TA), and AuNPs-TA (TA without plasma).

For scale-up, 50 mL of 0.2 mM HAuCl_4_ (five times the small-scale volume) was placed in a glass beaker, and 100 µL of TA stock was added to maintain the small-scale TA:Au molar ratio.

### 2.4. Characterization

We recorded UV–Vis absorption spectra on an Ocean Optics USB2000 spectrophotometer (Ocean Optics, Winter Park, FL, USA) using a quartz cuvette with a 10 mm path length. For in situ measurements, we treated 3 mL of the precursor solution in the same cuvette type directly with the plasma jet and recorded spectra every 20 s during treatment to track AuNP formation over time.

Scanning electron microscopy (SEM) images were obtained using a Hitachi S-4800 instrument (Hitachi High-Technologies Corporation, Tokyo, Japan). Transmission electron microscopy (TEM) images of the AuNPs-TA-Plasma sample were acquired on a JEM-2100 microscope (JEOL Ltd., Tokyo, Japan). Particle-size distributions were obtained by measuring more than 100 particles in TEM images using ImageJ (version 1.54, Java 8).

XPS spectra were acquired with a ULVAC-PHI 5000 VersaProbe II (ULVAC, Chigasaki, Kanagawa, Japan) [31] using a scanning monochromatic Al Kα X-ray beam (1486.6 eV) focused on a 100 µm wide spot at the surface. Because droplet drying on Si led to heterogeneous deposition, we performed measurements at three locations easily identified by scanning X-ray imaging (SXI). Survey spectra (pass energy of 117.4 eV) were obtained before and after high-resolution spectra (pass energy of 29.35 eV) to ensure that X-rays did not induce significant changes to the sample. The photoelectron takeoff angle was kept at 45° from the sample surface. For each measurement, we compensated for charging with a very low-energy electron beam, confirmed by the position of the sp^3^ C–C component in the C 1s spectra, all close to 285 ± 0.1 eV. Five and ten sweeps were acquired for the survey and high-resolution spectra, respectively.

### 2.5. SERS Measurements

For SERS measurements, we centrifuged 1.5 mL of each as-synthesized AuNP colloid at 5000 rpm for 10 min. The supernatant was removed, and the particles were redispersed in DI water to a total volume of 0.2 mL. We cut silicon wafers into small pieces, cleaned them with ethanol and DI water, and dried them with a nitrogen gun. An aliquot (10 µL) of each concentrated AuNPs suspension was drop-cast onto a wafer and dried at room temperature to form the SERS substrates. Then, 10 µL of the analyte solution was deposited onto the SERS substrates and dried at 60 °C before Raman analysis. We recorded Raman spectra with 532 nm excitation at approximately 8 mW and an acquisition time of 30 s; we repeated each measurement three times.

## 3. Results and Discussion

### 3.1. Effect of TA on the Reduction of HAuCl_4_

**Figure 3:**
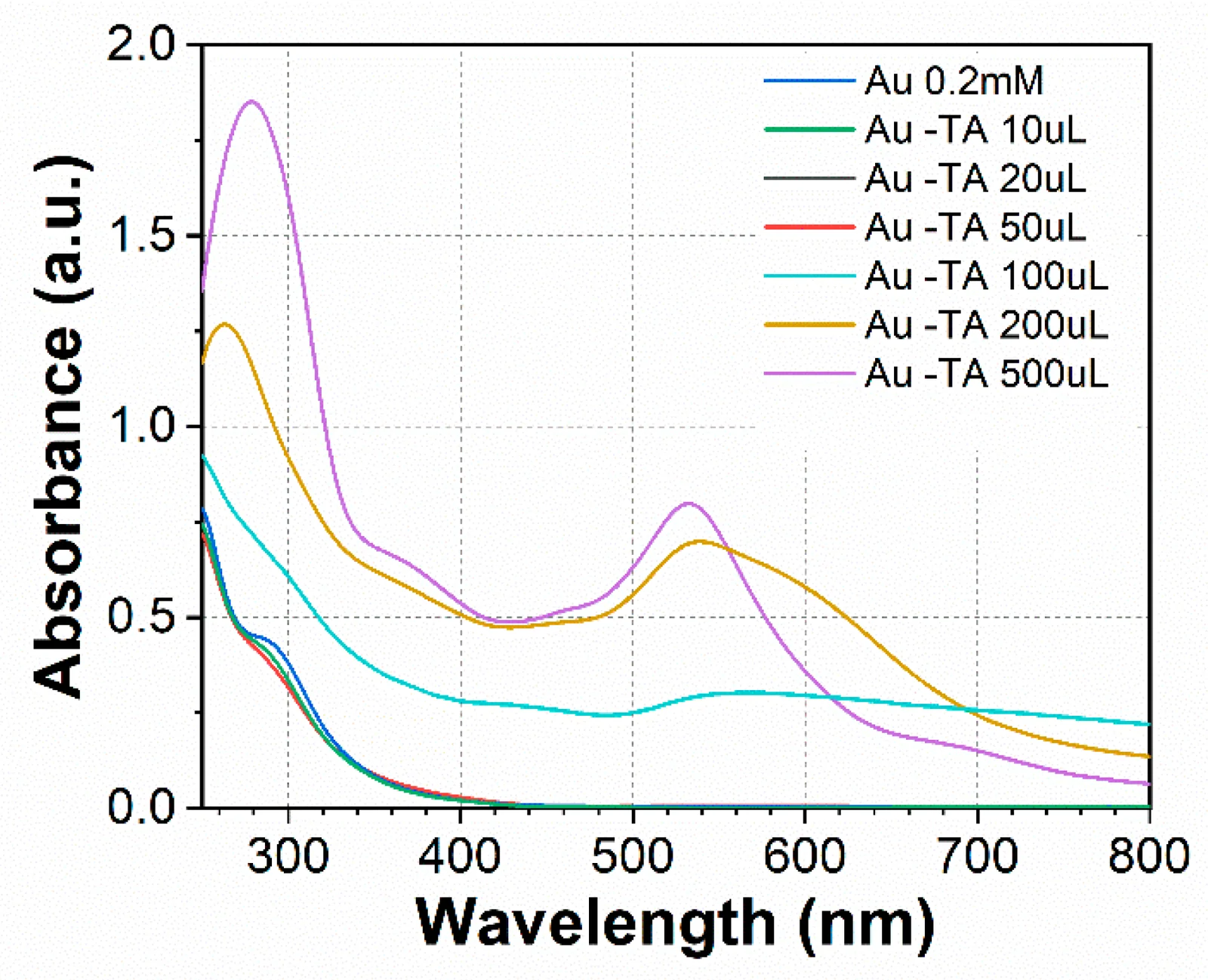
UV–Vis spectra of the plasma-free control: 0.2 mM HAuCl_4_ alone and with 10–500 µL of TA stock at room temperature after 10 min. A defined SPR band (531 nm) appears only at 500 µL.

We first evaluated TA’s reducing capacity by mixing 0.2 mM HAuCl_4_ with 10, 20, 50, 100, 200, and 500 µL of TA stock at room temperature without plasma treatment. The corresponding UV–Vis spectra are shown in Figure 3. All samples showed an intense absorption band in the UV region (270–320 nm), which increased with TA volume. However, this increase cannot be directly attributed to a higher concentration of unreduced Au^3+^. TA itself absorbs strongly in this region because of its phenolic groups, which overlap with the Au^3+^ ligand-to-metal charge-transfer band near 300 nm. Therefore, the progressive increase in UV absorbance primarily reflects TA’s increasing contribution to the overall spectrum, rather than directly measuring the remaining Au^3+^ concentration. A more reliable indicator of Au^3+^ reduction is therefore the appearance of the visible-region SPR band characteristic of metallic Au^0^ nanoparticles. At low TA volumes (10–50 µL), we observed no visible color change or SPR band, indicating that these TA doses were insufficient to induce appreciable Au^3+^ reduction and subsequent Au nanoparticle nucleation under room-temperature conditions. When the TA volume increased to 100–200 µL, a broad, relatively indistinct absorption feature appeared in the 500–600 nm region, suggesting the onset of Au^0^ nanoparticle formation. However, a clearly defined SPR band centered around 530 nm appeared only when the TA volume was increased further to 500 µL, confirming more pronounced formation of metallic Au^0^ nanoparticles.

These results show that TA alone can promote Au^3+^ reduction and Au^0^ nanoparticle formation under room-temperature conditions. Still, a relatively high TA dose is required to obtain a clearly identifiable SPR response. These observations motivate introducing plasma as an additional activation route, where plasma-generated reactive species can facilitate Au^3+^ reduction and promote more efficient AuNP formation, while TA can simultaneously stabilize the nanoparticles.

### 3.2. Effect of HAuCl_4_ Concentration

**Figure 4:**
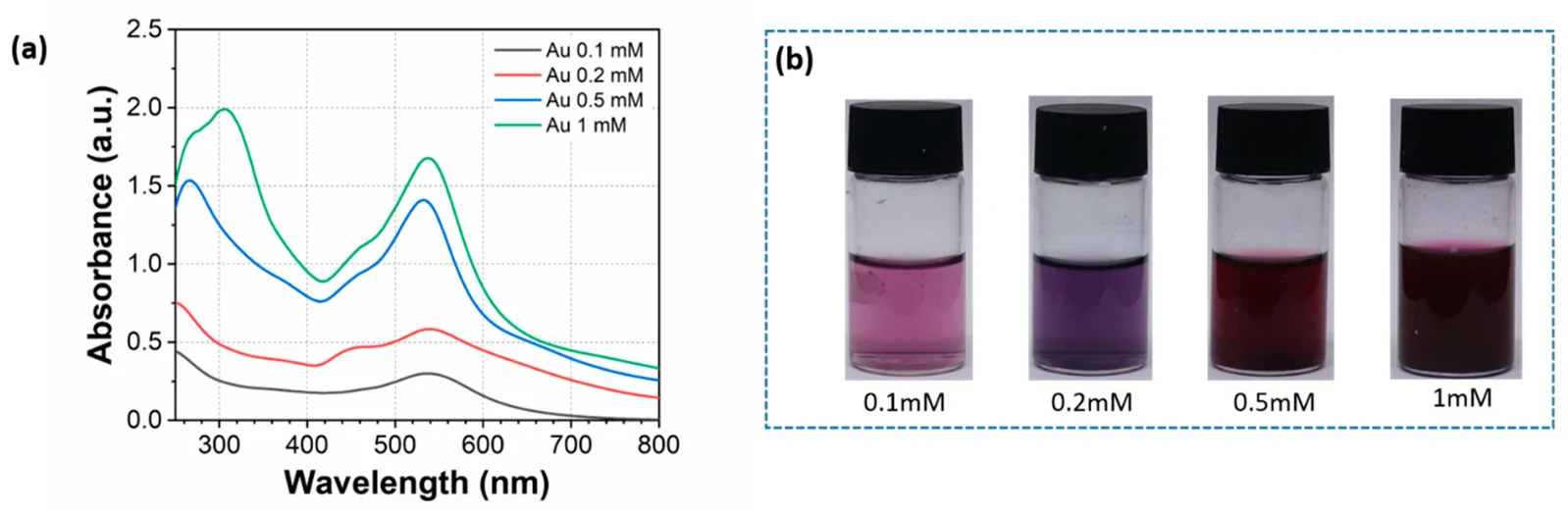
(**a**) UV–Vis spectra and (**b**) photographs of AuNPs synthesized by plasma treatment of 0.1, 0.2, 0.5, and 1 mM HAuCl_4_ without TA.

We first investigated the effect of HAuCl_4_ precursor concentration on AuNP formation in the absence of TA to assess how initial Au^3+^ concentration influences plasma-induced nanoparticle formation (Figure 4). We examined HAuCl_4_ concentrations of 0.1, 0.2, 0.5, and 1 mM under identical plasma treatment conditions. As shown in Figure 4, increasing the HAuCl_4_ concentration progressively changed the colloidal color from pale pink to violet and eventually to dark red, indicating increased formation of Au-containing nanoparticles. The corresponding UV–Vis spectra showed visible-region absorption features, with the SPR response becoming more pronounced as precursor concentration increased. At 0.1 and 0.2 mM, the visible-region absorption intensity was relatively low, indicating limited AuNP formation. Increasing the precursor concentration to 0.5 mM produced a stronger SPR band around 530 nm, accompanied by increased spectral broadening. Further increasing the precursor concentration to 1 mM produced an intense but broad absorption profile in the visible region, along with a dark red colloidal solution, suggesting increased nanoparticle formation but also a greater tendency toward particle growth and aggregation. The broadening of the SPR band at higher precursor concentrations indicates a less uniform nanoparticle population, likely due to increased Au^3+^ availability during plasma-induced reduction. These results demonstrate that precursor concentration strongly influences both the amount and optical characteristics of the plasma-synthesized AuNPs. Because we conducted the experiments without a stabilizing agent, increasing the Au^3+^ concentration beyond a certain level promotes particle growth and reduces colloidal uniformity.

**Figure 5:**
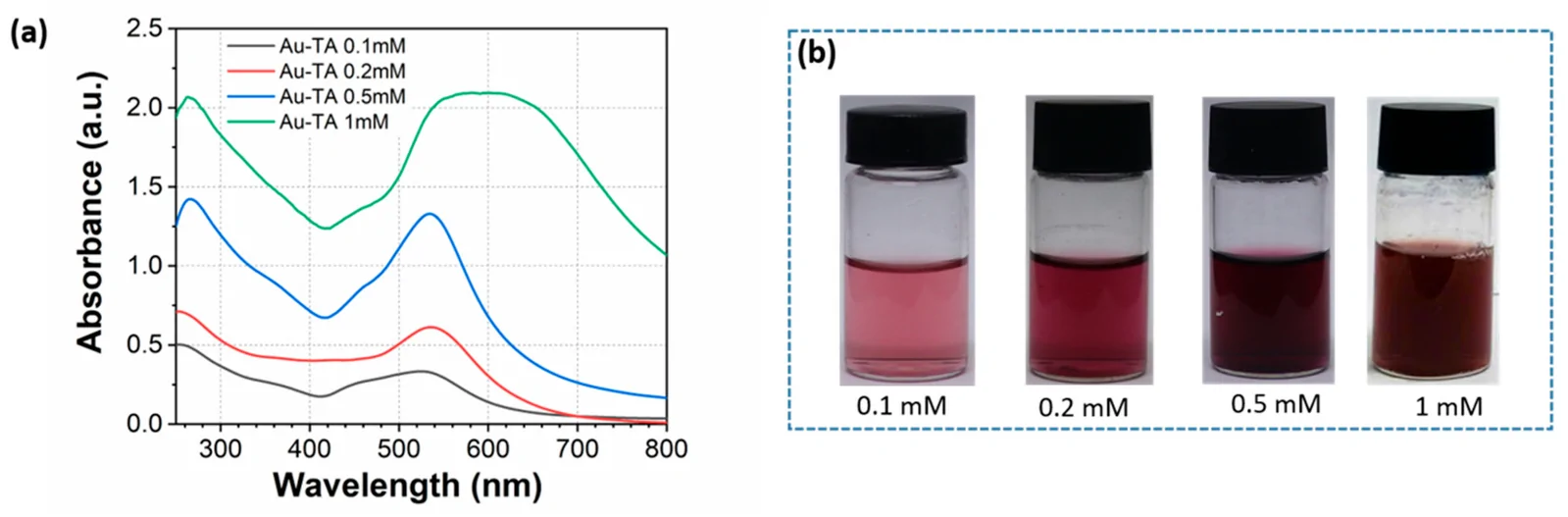
(**a**) UV–Vis spectra and (**b**) photographs of AuNPs synthesized from 0.1, 0.2, 0.5 and 1 mM HAuCl_4_ by plasma treatment with 20 µL TA.

Therefore, to improve colloidal stability and further determine the suitable precursor concentration, we subsequently investigated the same HAuCl_4_ concentration range with a fixed TA volume of 20 µL (Figure 5). At 0.1 mM HAuCl_4_, a characteristic SPR peak appeared at approximately 530 nm, confirming the formation of metallic Au^0^ nanoparticles; however, the relatively low absorbance indicates a lower nanoparticle concentration due to the limited precursor concentration. Increasing the HAuCl_4_ concentration to 0.2 mM increased absorbance and produced a relatively sharp SPR peak, indicating efficient nanoparticle formation and good colloidal stability. Further increasing the precursor concentration to 0.5 and 1 mM broadened the SPR peak, suggesting increased particle size. This behavior can be attributed to the increased Au^3+^ relative to the fixed TA concentration, lowering the TA:Au molar ratio and reducing surface passivation effectiveness. After plasma treatment, the 0.5 and 1 mM colloids gradually precipitated, indicating low stability.

### 3.3. Effect of TA Dose

**Figure 6:**
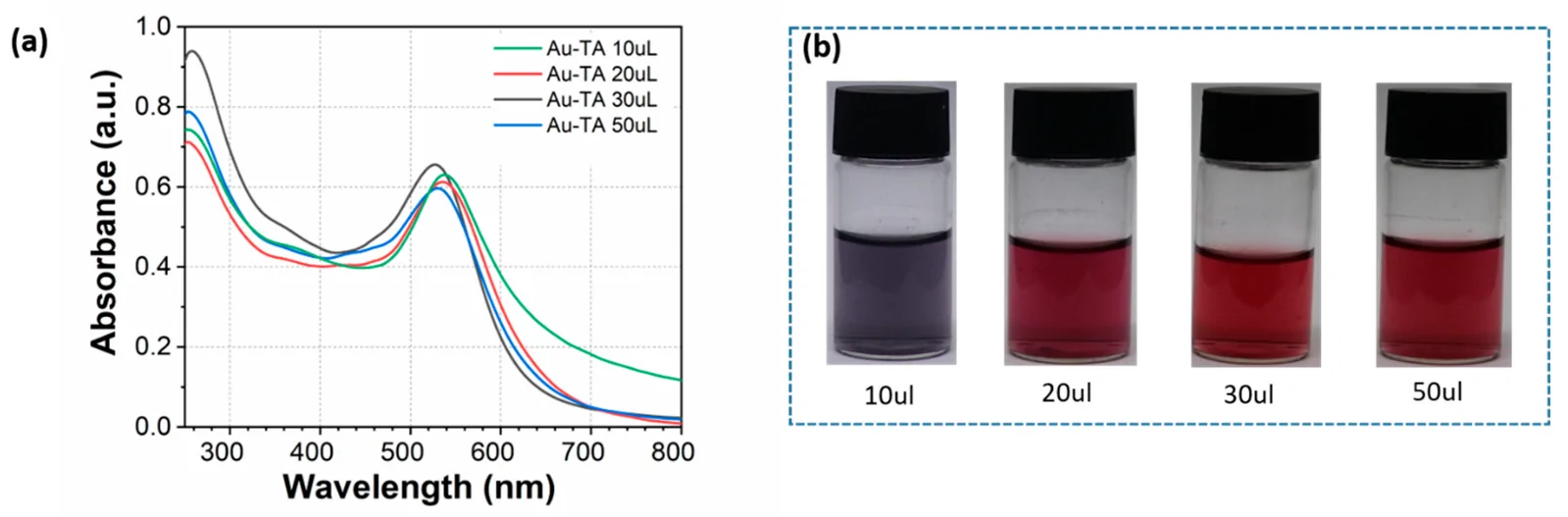
(**a**) UV–Vis spectra and (**b**) photographs of AuNPs synthesized with 10, 20, 30, and 50 µL TA (0.2 mM HAuCl_4_, 10 min).

We further investigated the effect of TA volume on AuNP formation while maintaining the HAuCl_4_ concentration at 0.2 mM and the plasma irradiation time at 10 min. We evaluated TA volumes of 10, 20, 30, and 50 µL, and Figure 6 presents the corresponding UV–Vis absorption spectra and photographs of the colloidal solutions. At 10 µL TA, the colloidal solution appeared slightly violet and showed a broad absorption profile extending beyond 600 nm, indicating the formation of relatively large, partially aggregated nanoparticles. This behavior suggests that the amount of TA was insufficient to effectively passivate the nanoparticle surfaces, thereby allowing continued particle growth and aggregation during plasma treatment. When the TA volume increased to 20 and 30 µL, the broad absorption profile narrowed considerably, with a distinct SPR band centered at approximately 530 nm. Meanwhile, the solution color changed from violet-blue to the characteristic ruby red associated with well-dispersed AuNPs. These changes indicate that increasing the TA amount improved surface stabilization and suppressed excessive particle growth and aggregation. Further increasing the TA volume to 50 µL caused no significant change in either the SPR peak position or spectral width compared with the 20 and 30 µL samples, suggesting that TA stabilization had reached an approximately saturated level under the investigated conditions.

### 3.4. Effect of Plasma Irradiation Time

**Figure 7:**
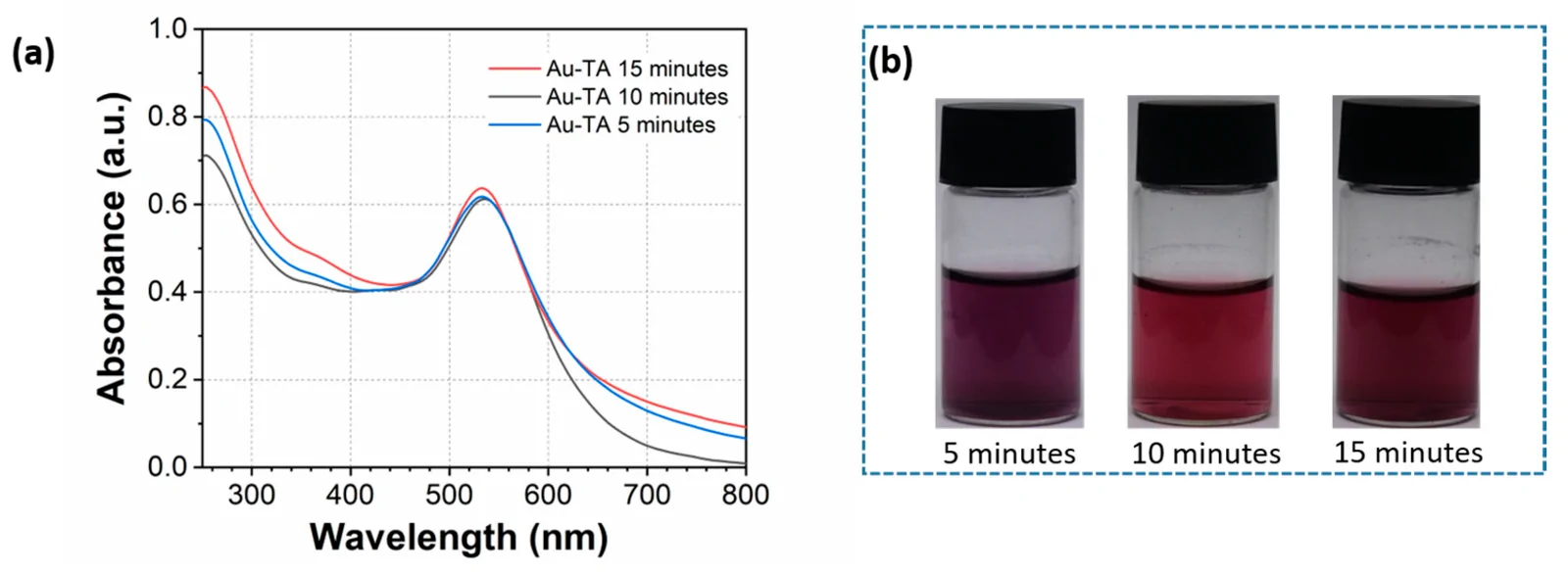
(**a**) UV–Vis spectra and (**b**) photographs of AuNPs-TA-Plasma after 5, 10, and 15 min of plasma treatment (0.2 mM HAuCl_4_, 20 µL TA).

After investigating the HAuCl_4_ precursor concentration and TA volume, we examined the effect of plasma irradiation time on AuNP formation by varying the treatment time from 5 to 15 min while keeping the HAuCl_4_ concentration at 0.2 mM and the TA volume at 20 µL. Figure 7 shows the corresponding UV–Vis absorption spectra and photographs of the colloidal solutions. All three conditions produced a characteristic SPR band at approximately 530 nm and a ruby-red colloidal color, confirming successful AuNP formation. At 5 min, we observed a relatively weak, broad SPR band and lower absorbance, indicating that the plasma treatment was insufficient to fully reduce Au^3+^ and form nanoparticles efficiently. Increasing the irradiation time to 10 min increased absorbance and produced a relatively sharp SPR band, indicating a higher yield of AuNPs with a relatively narrow size distribution.

In contrast, extending the treatment time to 15 min did not further increase the absorbance. Instead, the SPR band slightly broadened and absorbance decreased slightly, suggesting that prolonged plasma exposure may promote further particle growth and aggregation, reducing colloidal uniformity. These results indicate that the plasma irradiation time plays an important role in balancing Au^3+^ reduction, nanoparticle nucleation, and subsequent particle growth. A 5 min treatment time appears to be insufficient for efficient nanoparticle formation, whereas excessive plasma exposure may favor particle growth and aggregation.

### 3.5. Effect of Argon Flow Rate

**Figure 8:**
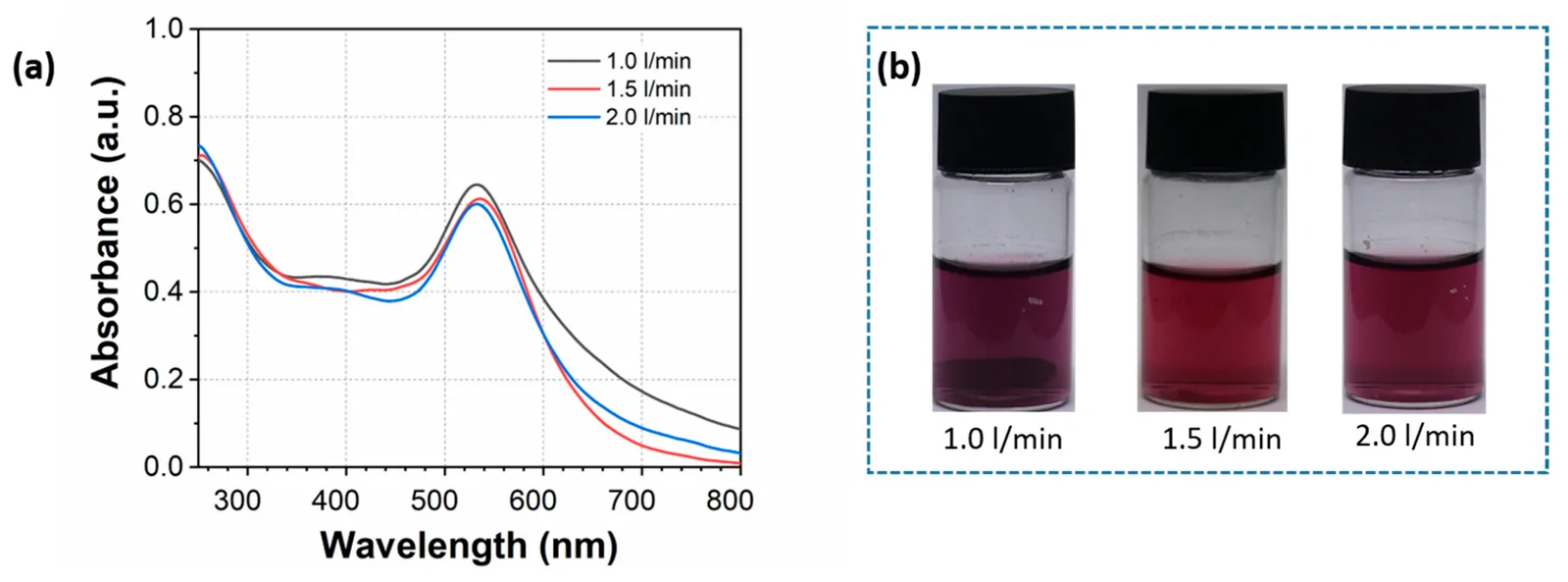
(**a**) UV–Vis spectra and (**b**) photographs of AuNPs-TA-Plasma synthesized at Ar flow rates of 1.0, 1.5, and 2.0 L·min^−1^.

We then investigate the effect of the Ar gas flow rate on AuNP formation (Figure 8). We varied the Ar flow rate from 1.0 to 2.0 L min-1 while keeping the HAuCl_4_ concentration at 0.2 mM, TA volume at 20 µL, and plasma irradiation time at 10 min. Figure 8 shows the corresponding UV–Vis absorption spectra and photographs of the colloidal solutions. All three conditions produced a well-defined SPR band at approximately 530 nm, accompanied by the characteristic ruby-red color, confirming successful AuNP formation over the flow rate range investigated. The sample prepared at a gas flow rate of 1.5 L min-1 showed the narrowest SPR band, indicating more uniform AuNP formation. At 1.0 L min^−1^, the plasma plume barely reached the solution surface. At 2.0 L min^−1^, the gas flow caused the solution to splash, losing some liquid and reducing the product volume.

### 3.6. Effect of pH

**Figure 9:**
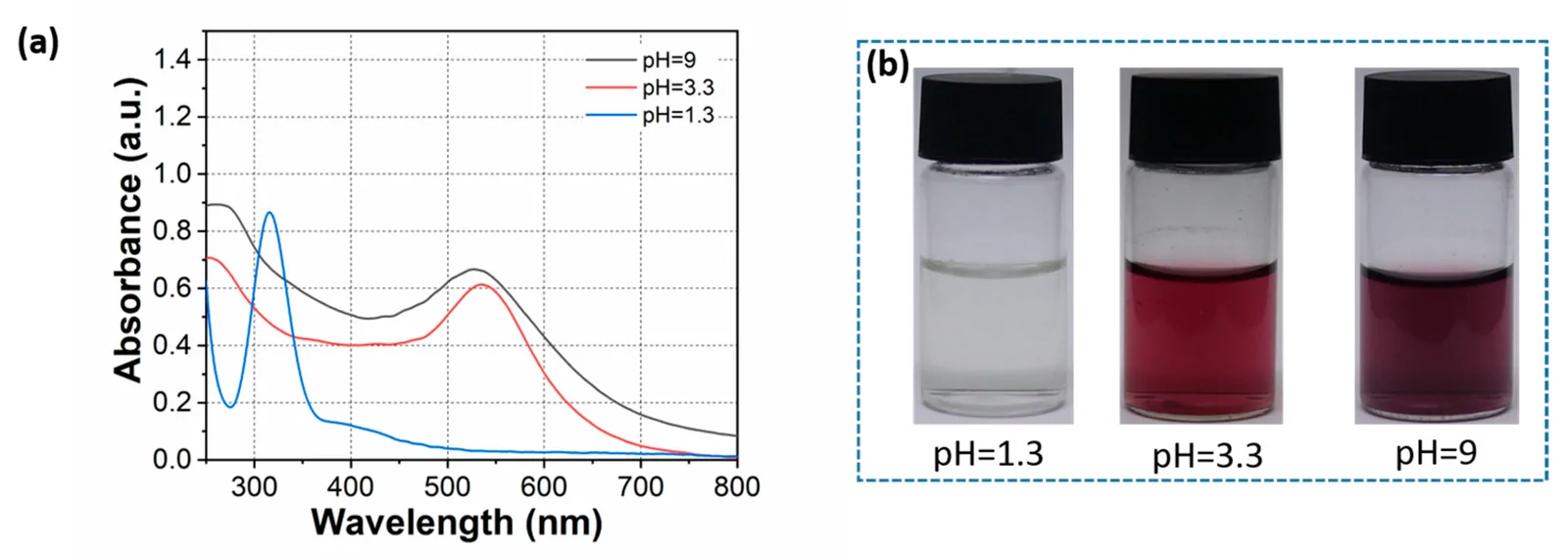
(**a**) UV–Vis spectra and (**b**) photographs of AuNPs-TA-Plasma synthesized at initial pH values of 1.3, 3.3, and 9.

The initial solution pH strongly affected AuNP formation and their optical properties (Figure 9). At pH 1.3, the solution remained nearly colorless, and no distinct SPR band appeared even after 10 min of plasma treatment, indicating strongly suppressed AuNP formation. This behavior can be attributed to the strongly protonated state of the phenolic groups in TA, which limits their electron-donating ability and contribution to Au^3+^ reduction. In addition, the high proton concentration may reduce the availability of plasma-generated reducing species, particularly solvated electrons, further suppressing Au^3+^ reduction and subsequent AuNP nucleation [32]. For the intrinsic pH of HAuCl_4_-TA (3.3), a distinct SPR band appeared at approximately 540 nm with a characteristic ruby-red color. Although TA remains predominantly protonated at this pH, the less acidic environment favors the combined action of plasma-generated reducing species and TA, resulting in

Efficient Au^3+^ reduction, nucleation, and surface stabilization. At pH 9, greater TA deprotonation enhances its electron-donating ability and interaction with the AuNP surface. The SPR band therefore became broader with higher SPR intensity. The results suggest that increasing pH to 9 could increase the AuNP formation rate but may reduce control over particle uniformity.

### 3.7. Scale-Up of the Plasma–TA Synthesis

**Figure 10:**
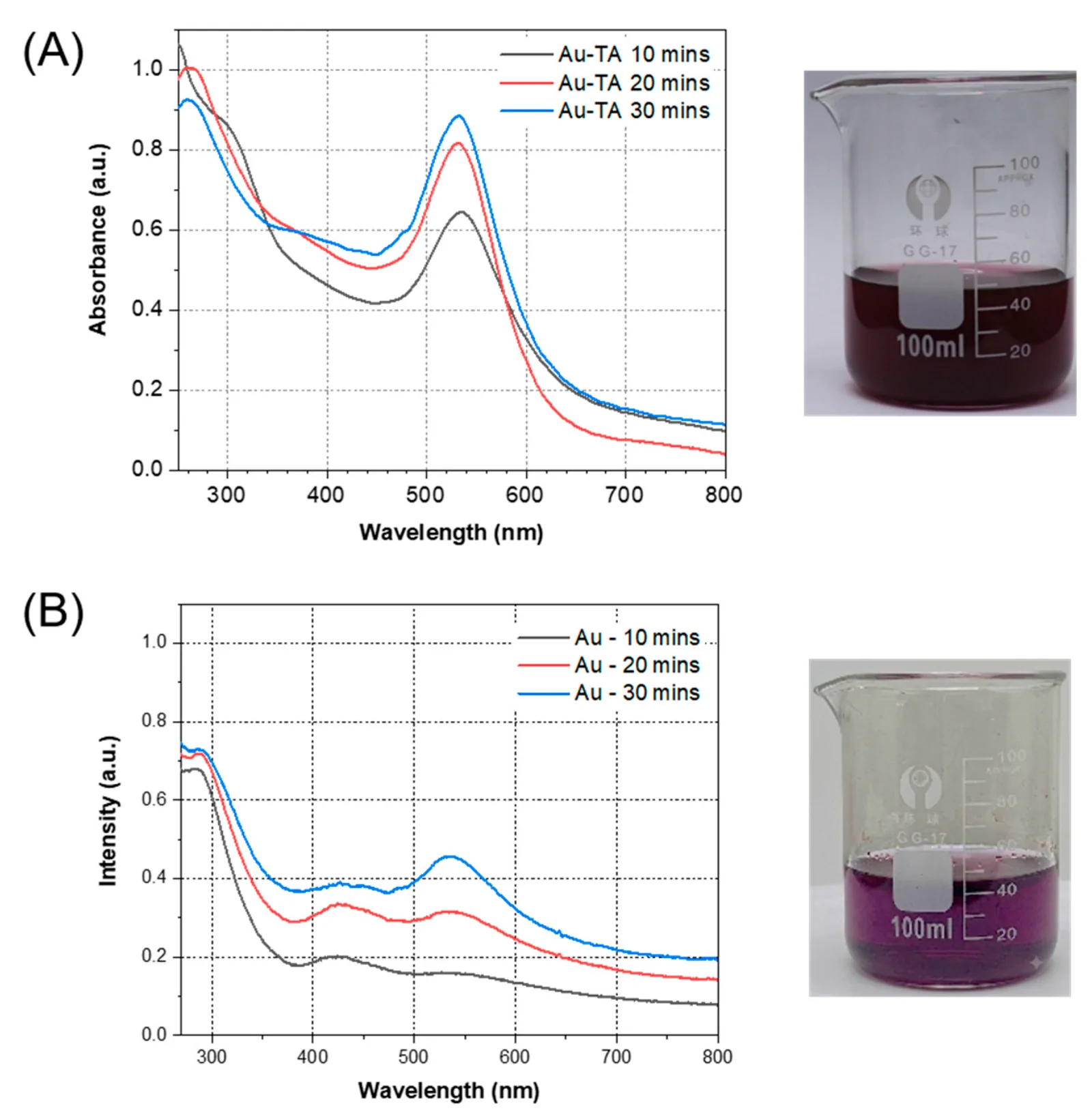
Plasma synthesis in a 50 mL batch (**A**) with 100 µL TA (TA:Au ≈ 0.01) and (**B**) without TA. UV–Vis spectra after 10, 20, and 30 min of plasma treatment, and photographs of the colloids after 30 min.

Plasma–liquid reactions are confined to a thin interfacial layer, so plasma-assisted nanoparticle syntheses are usually limited to small volumes, typically less than 10 mL [33]. To test a larger volume, we treated 50 mL of 0.2 mM HAuCl_4_ with 100 µL of TA or without TA (Figure 10). The TA: Au ratio remained the same as in the 10 mL experiments. The larger volume also required longer treatment, up to 30 min. This is expected at a fixed plasma–liquid contact area, because the reducing species are produced only at the interface and must be carried into the bulk by stirring. The intensity of the SPR peak of AuNPs-TA-Plasma (about 0.9) is significantly higher than that of AuNPs Plasma (<0.5). This experiment demonstrates the potential to scale up this approach.

### 3.8. Characterization of AuNPs-TA-Plasma

**Figure 11:**
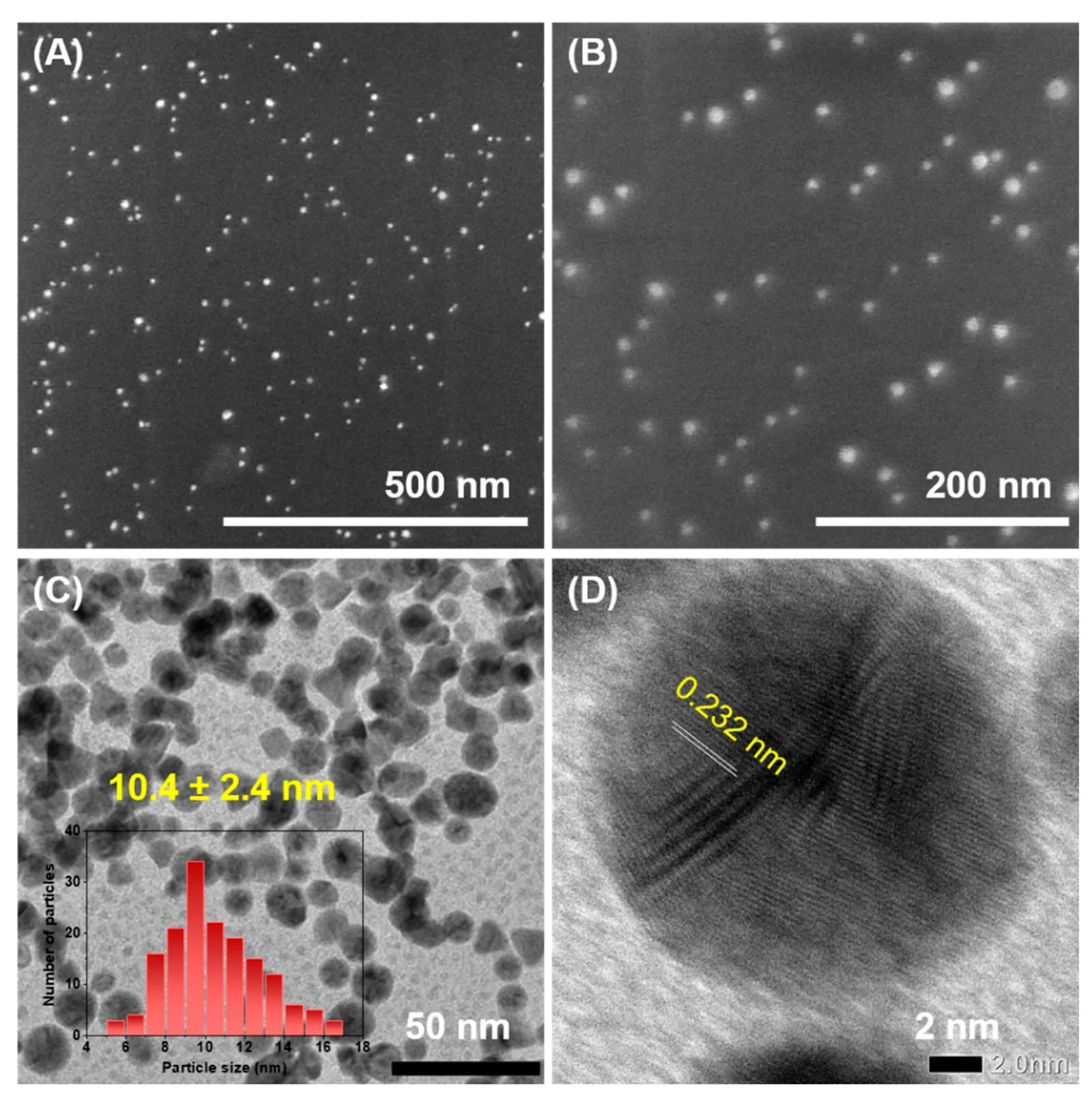
(**A**,**B**) SEM images of the optimized AuNPs-TA-Plasma at two magnifications (scale bars 500 and 200 nm); (**C**) TEM image (scale bar 50 nm) with the particle-size histogram (inset; 10.4 ± 2.4 nm); (**D**) HRTEM image (scale bar 2 nm) showing lattice fringes with d = 0.232 nm, indexed to Au(111).

We selected AuNPs-TA-Plasma prepared with 10 mL of 0.2 mM HAuCl_4_ and 20 µL TA (1 mM) for several characterizations. Figure 11A,B demonstrate the SEM of AuNPs-TA-Plasma at different magnifications, revealing small, discrete, and uniform particles. TEM image (Figure 11C) showed predominantly quasi-spherical particles, some with faceted or slightly elongated outlines. Their mean diameter was estimated to be 10.4 ± 2.4 nm. The particles are considerably smaller than the plasma-synthesized AuNPs obtained with similar argon jets with stabilizer-free (45 nm) [21], polydopamine-coated [19], or PEG-coated (32.5 ± 10.1) [34]. HRTEM resolved continuous lattice fringes with an interplanar spacing of 0.232 nm (Figure 11D), close to the Au(111) spacing of face-centered cubic gold (0.235 nm). No distinct organic shell is visible around the particles.

**Figure 12:**
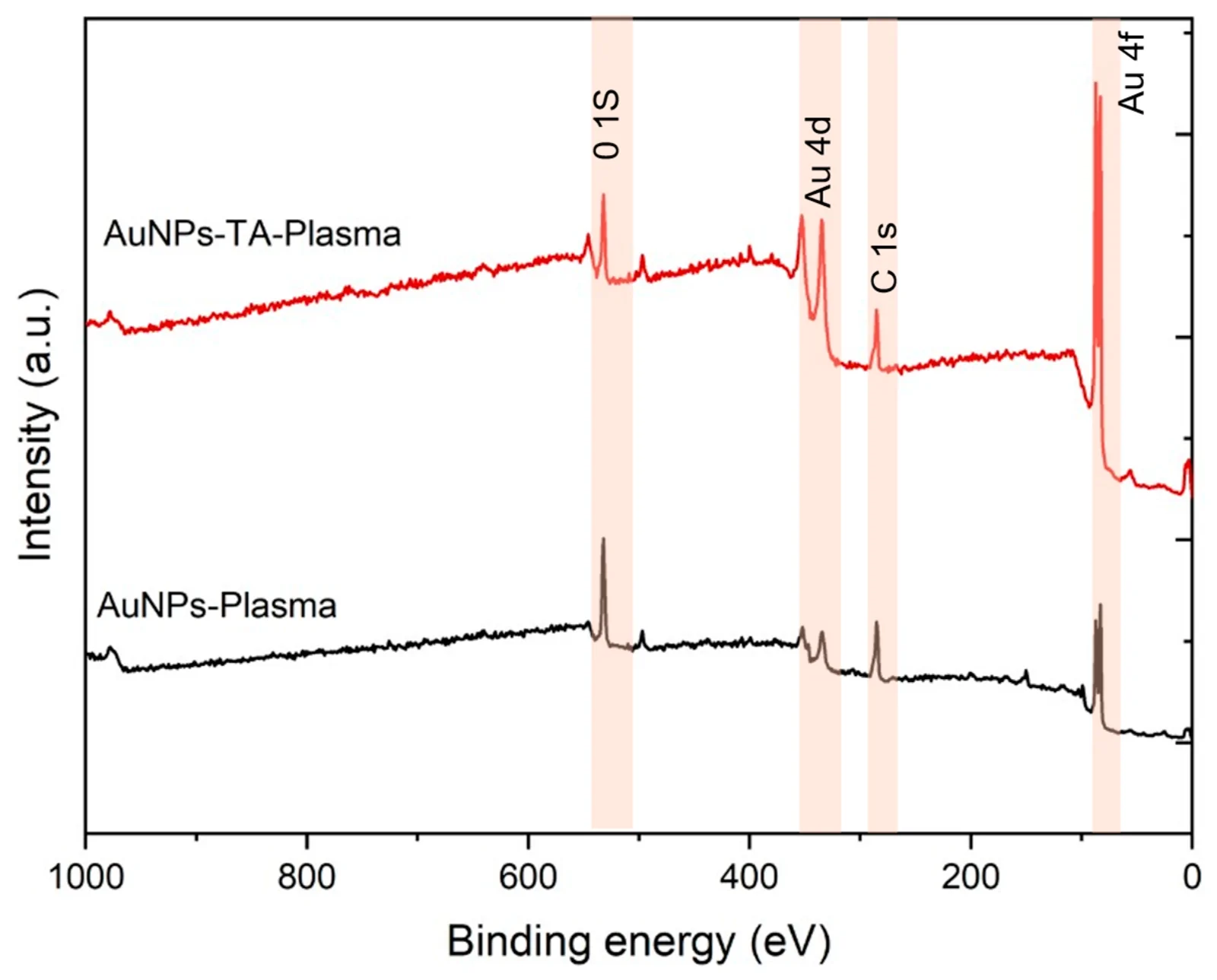
XPS survey spectra of AuNPs-TA-Plasma (red curve) and AuNPs-Plasma (black curve).

**Figure 13:**
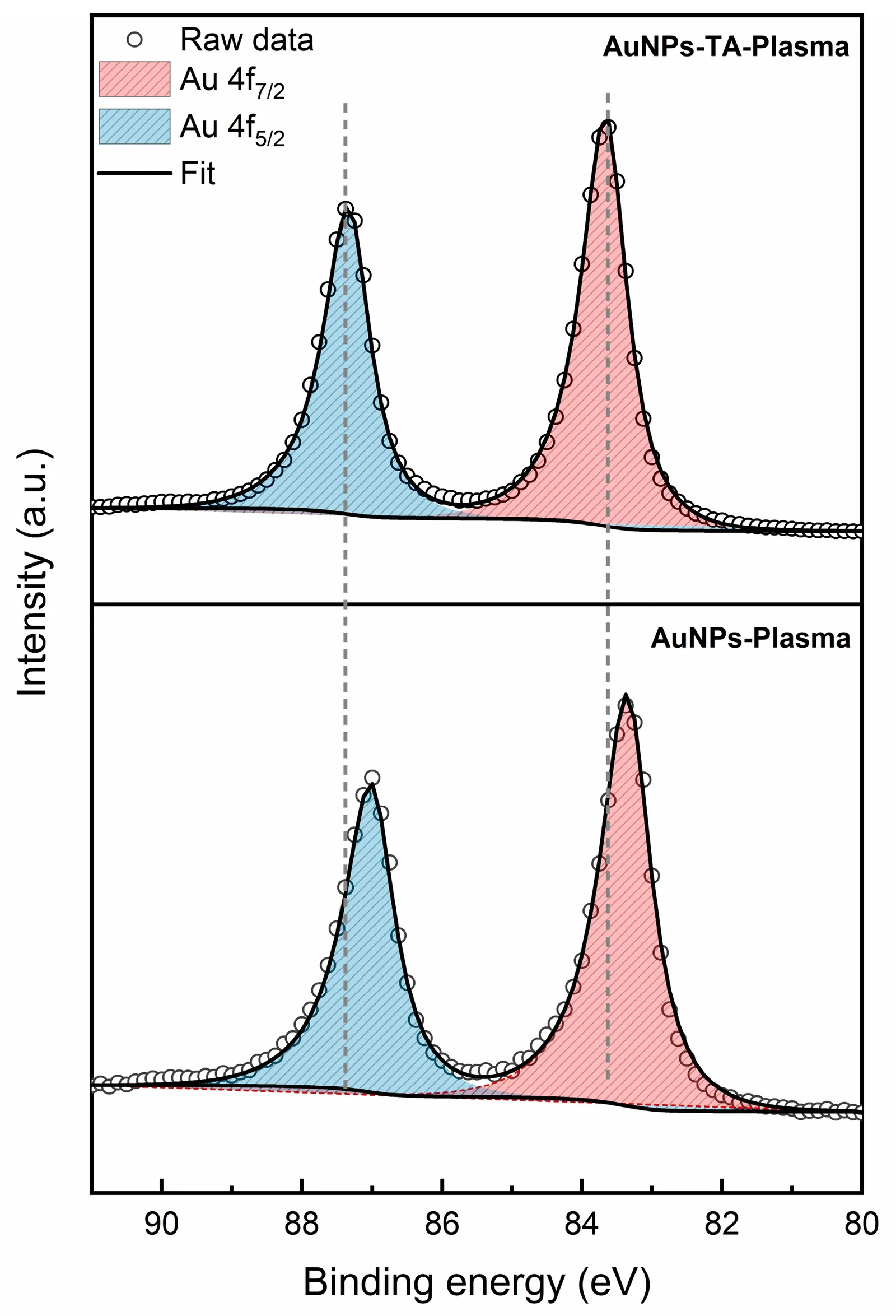
High-resolution Au 4f XPS spectra of AuNPs-TA-Plasma and AuNPs-Plasma.

Figure 12 compares the XPS survey spectra of AuNPs-TA-Plasma (red curve) and AuNPs-Plasma (black curve). The spectra show photoelectron peaks from Au 4f (84/88 eV), Au 4d (335/353 eV), C 1s (285 eV), O 1s (532 eV), and Au 4p (546 eV) [35]. XPS showed that the gold in both plasma-synthesized samples is metallic Au^0^, but that the Au 4f peaks of AuNPs-TA-Plasma are shifted by 0.3 eV to higher binding energy relative to those of AuNPs-Plasma (Figure 13). This could be attributed to the existence of TA at the surface of AuNPs-TA-Plasma.

### 3.9. Investigation of the Reaction Mechanism

**Figure 14:**
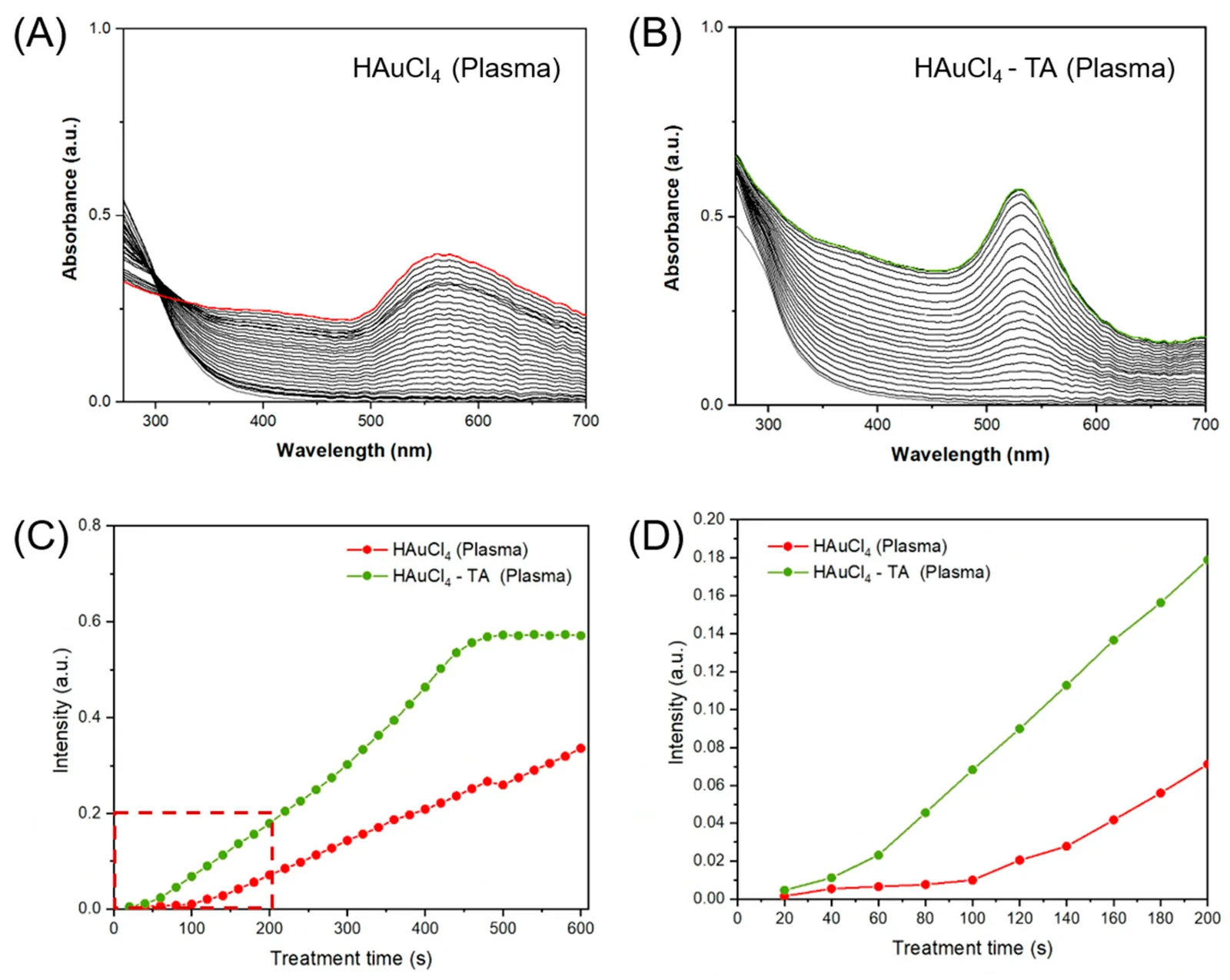
In situ UV–Vis spectra recorded during plasma treatment of HAuCl_4_ (**A**) without TA and (**B**) with TA; (**C**) absorbance at the SPR maximum versus treatment time; (**D**) enlarged view of the first 200 s.

To test whether low-dose TA shortens the induction period of plasma reduction, particle formation was investigated by in situ UV–Vis spectroscopy during treatment of HAuCl_4_ with and without TA (Figure 14). These measurements used a smaller volume (3 mL) in a 1 mm light-path cuvette; therefore, the absorbance values are not directly comparable with those of the experiments above. Without TA (Figure 14A), the SPR band developed slowly and remained broad, with its maximum near 565 nm, which indicates relatively large or aggregated particles. The absorbance at the band maximum remained near zero for the first 100 s of treatment and increased only afterward (Figure 14C,D). The absorbance below 310 nm, where the ligand-to-metal charge-transfer band of AuCl_4_^−^ absorbs, decreased progressively during treatment, and the spectra crossed near 310 nm. Au^3+^ was therefore consumed while no plasmonic particles were yet detectable. This is the behavior expected for the induction period discussed above, as plasma-generated electrons first convert Au^3+^ to colorless Au^+^ species, and Au^3+^ competes with Au^+^ for the reducing equivalents. Detectable nuclei form only after a substantial fraction of the Au^3+^ has been converted. After the induction period, the SPR absorbance increased steadily and did not plateau by 600 s, so reduction was still incomplete at the end of the measurement.

In the presence of TA (Figure 14B), a narrow SPR band at 530 nm appeared from the beginning of treatment. Its absorbance was measurable immediately after plasma ignition and rose steadily. The induction period was much less pronounced than that of the TA-free solution. The absorbance plateaued at about 480 s, indicating that particle formation was essentially complete. The band remained narrow and at the same position throughout, consistent with the growth of small, well-dispersed particles rather than aggregation. The early appearance of particles must therefore result from the combined action of TA and plasma rather than from reduction by TA alone. These results provide direct evidence that low-dose TA shortens the induction period of plasma-driven Au reduction.

**Figure 15:**
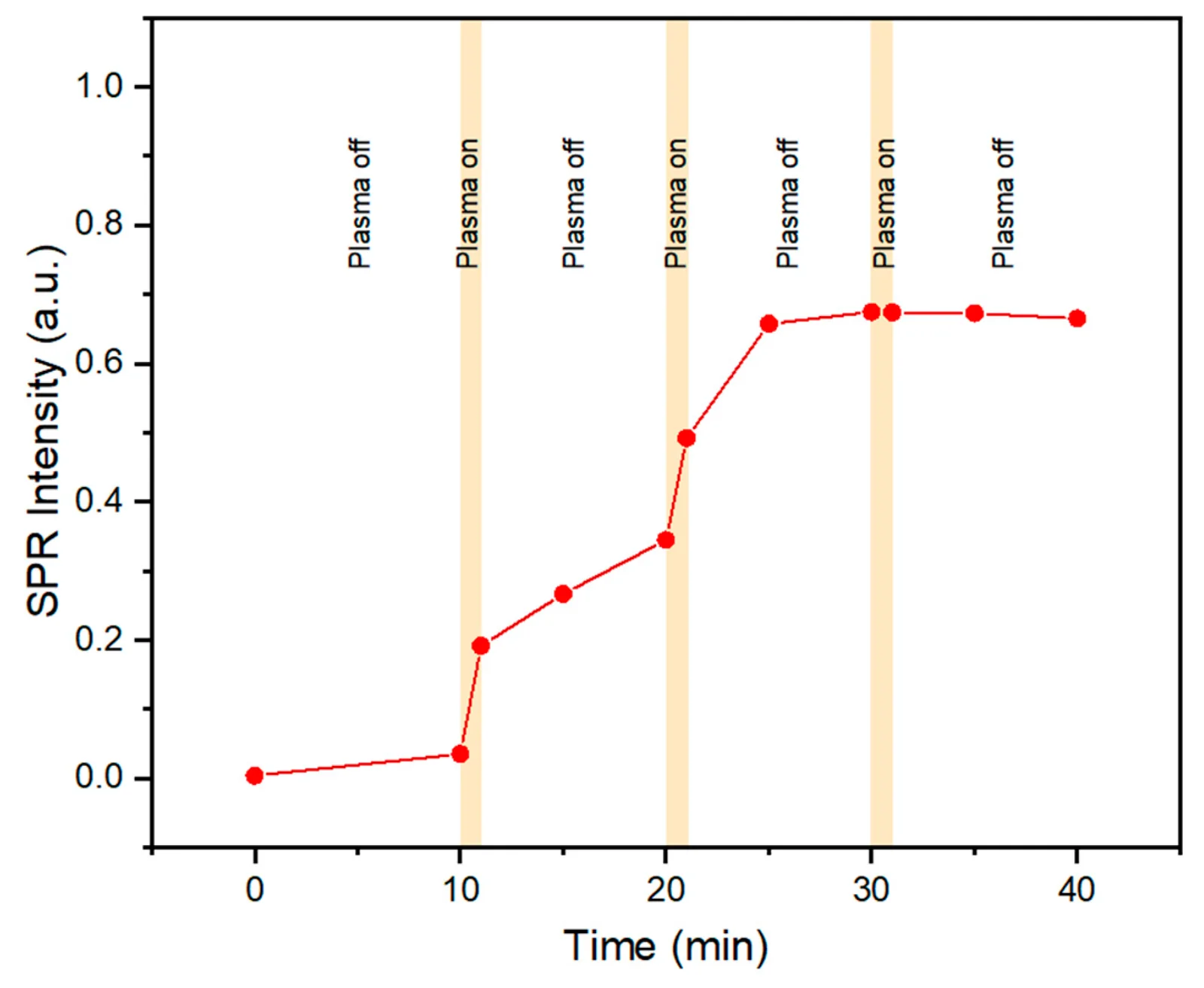
SPR absorbance of 0.2 mM HAuCl_4_ with TA versus time, during intermittent plasma treatment (1-min pulses at 10, 20, and 30 min; shaded bars). We kept the solution stirred at room temperature without plasma between pulses.

To test whether reduction continues after the discharge is switched off, we exposed the HAuCl_4_–TA solution to short 1-min plasma pulses, separated by 9 min without plasma (Figure 15). During the first 10 min without plasma, the SPR absorbance increased only slightly, from 0 to 0.03. This result confirms that low-dose TA alone reduces Au^3+^ very slowly and forms few, if any, nuclei (Section 3.1). A single 1-min plasma pulse raised the absorbance sharply to 0.19. After the discharge was switched off, the absorbance intensity continued to increase, reaching 0.26 at 15 min and 0.34 at 20 min, significantly faster than after the first pulse. A second pulse again induces an increase to 0.49, followed by even faster growth without plasma, reaching 0.65 at 25 min. After that, the absorbance leveled off. A third pulse at 30 min had almost no effect, and the value stayed at 0.66 until 40 min, which indicates that the gold precursor was essentially consumed. Each pulse therefore has two effects. During discharge, plasma-generated electrons reduce Au^3+^ and Au^+^ directly at the plasma–liquid interface and produce a burst of nuclei, causing the immediate jump in absorbance. After the discharge, reduction continues in the bulk solution without plasma.

Taken together, the in situ and intermittent plasma exposure experiments indicate that low-dose TA accelerates plasma reduction kinetically rather than by supplying reducing equivalents. Plasma provides most of the reducing power, and TA therefore needs to be present only in amounts sufficient to stabilize the nuclei. This can explain why the TA dose can be much lower than in conventional green syntheses. Table 1 compares previous studies of TA-mediated green synthesis of AuNPs with the corresponding TA:Au molar ratio [36]. In this work, the synthesis of AuNPs-TA-Plasma can be carried out at room conditions by plasma treatment with a low TA: Au molar ratio of 0.01:1, without the need of additional chemicals.

To further clarify the roles of TA and plasma-generated species in AuNP formation after the discharge is switched off, we performed three control experiments (Figures S1 and S2). As shown in Figure S1, adding HAuCl_4_ to plasma-treated water did not produce a distinct SPR band in the visible region, indicating that plasma-treated water alone was insufficient to generate a detectable population of plasmonic AuNPs under the conditions investigated.

In contrast, the SPR absorbance increased when HAuCl_4_ was added to TA that had been pretreated with plasma in water (Figure S2). After plasma treatment, the absorbance of TA at 270–280 nm decreased markedly, suggesting that plasma-generated reactive species oxidized and/or partially degraded TA into smaller species. After adding HAuCl4, the SPR band at about 540 nm increased from approximately 0.60 after 10 min to 0.85 after 20 min. This result indicates that AuNP formation and/or growth can proceed after discharge stops in a solution containing TA used during plasma treatment.

### 3.10. Demonstration of SERS Performance

**Figure 16:**
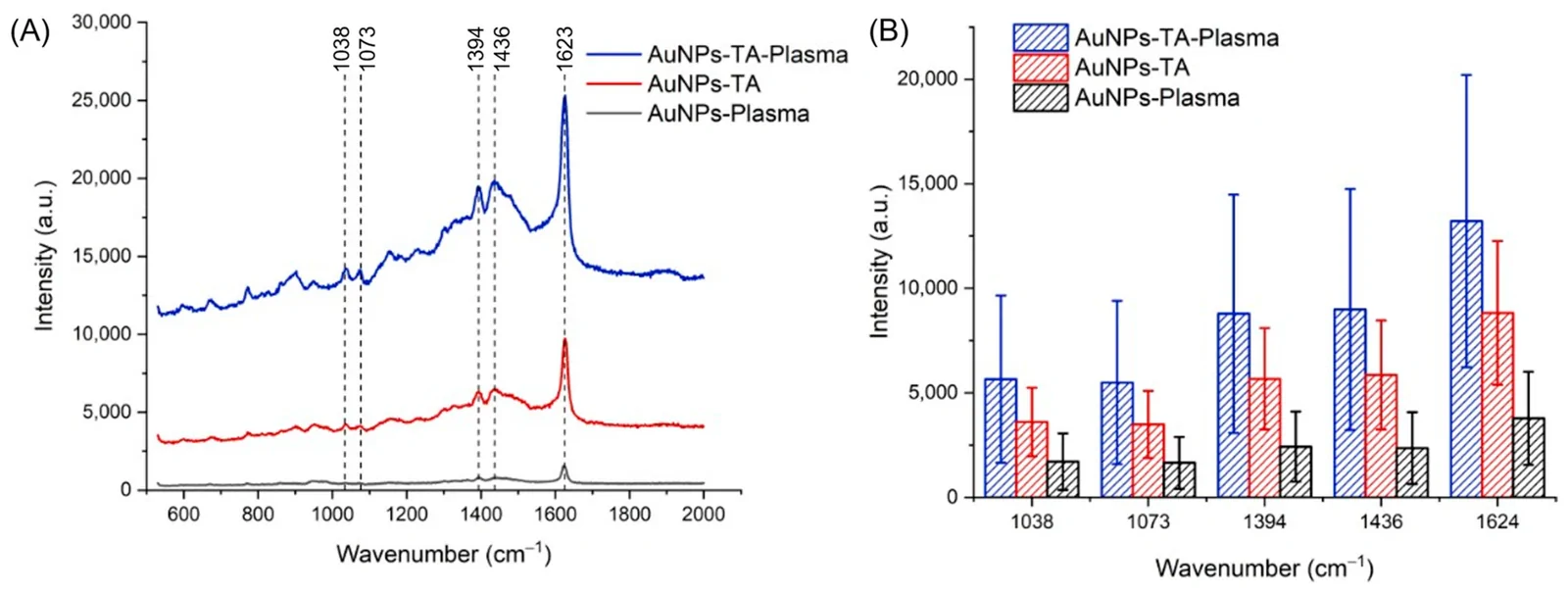
SERS spectra of MB on drop-cast substrates of AuNPs-TA-Plasma, AuNPs-TA, and AuNPs-Plasma. (**A**) Representative spectra, shown without baseline correction; (**B**) mean intensities of the 1038, 1073, 1394, 1436, and 1624 cm^−1^ bands.

We investigate the sensing performance of the AuNPs-TA-Plasma product prepared with 20 µL TA to evaluate its potential as a SERS substrate. For comparison, we prepare AuNPs-Plasma, made by the same plasma treatment without TA, and AuNPs-TA, made without plasma at the TA dose required for reduction by TA alone (500 µL). Figure S3 shows the UV-Vis spectra of these samples. We concentrated each colloid by centrifugation, drop-cast it onto Si, and loaded it with 10 µL of 100 ppm MB. All three substrates gave the characteristic MB spectrum (Figure 16A). The strongest band appeared at 1624 cm^−1^ (C–C ring stretching), accompanied by bands at 1436 and 1394 cm^−1^ (asymmetric and symmetric C–N stretching) and weaker bands at 1038 and 1073 cm^−1^ (C–H in-plane bending). These results suggest that AuNPs-TA-Plasma provides stronger SERS enhancement than AuNPs-TA and AuNPs-Plasma under identical analytical conditions (Figure 16B).

**Figure 17:**
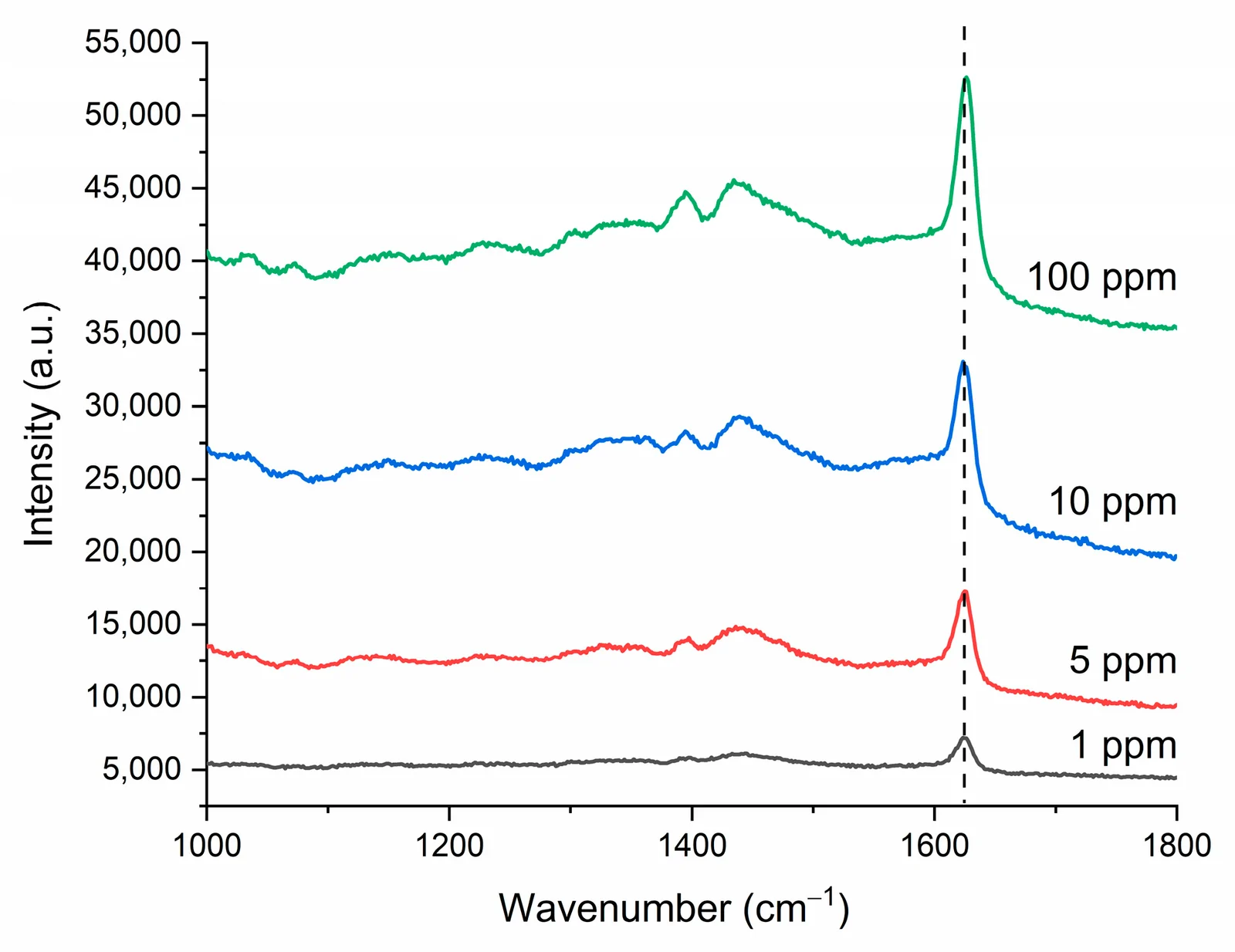
SERS spectra of MB at 1, 5, 10, and 100 ppm on AuNPs-TA-Plasma substrates (532 nm excitation). The dashed line marks the 1624 cm^−1^ band.

To assess the AuNPs-TA-Plasma substrate’s sensitivity, we recorded SERS spectra of MB at concentrations from 1 to 100 ppm (Figure 17). The characteristic MB band at 1624 cm^−1^ (C–C ring stretching) was clearly detected at all concentrations, including 1 ppm. The weaker bands at 1394 and 1436 cm^−1^ became distinguishable from 5 ppm upward. The band position did not change with concentration, indicating that MB adsorbs in a similar configuration across the range studied. The 1624 cm^−1^ band intensity increased with MB concentration.

**Figure 18:**
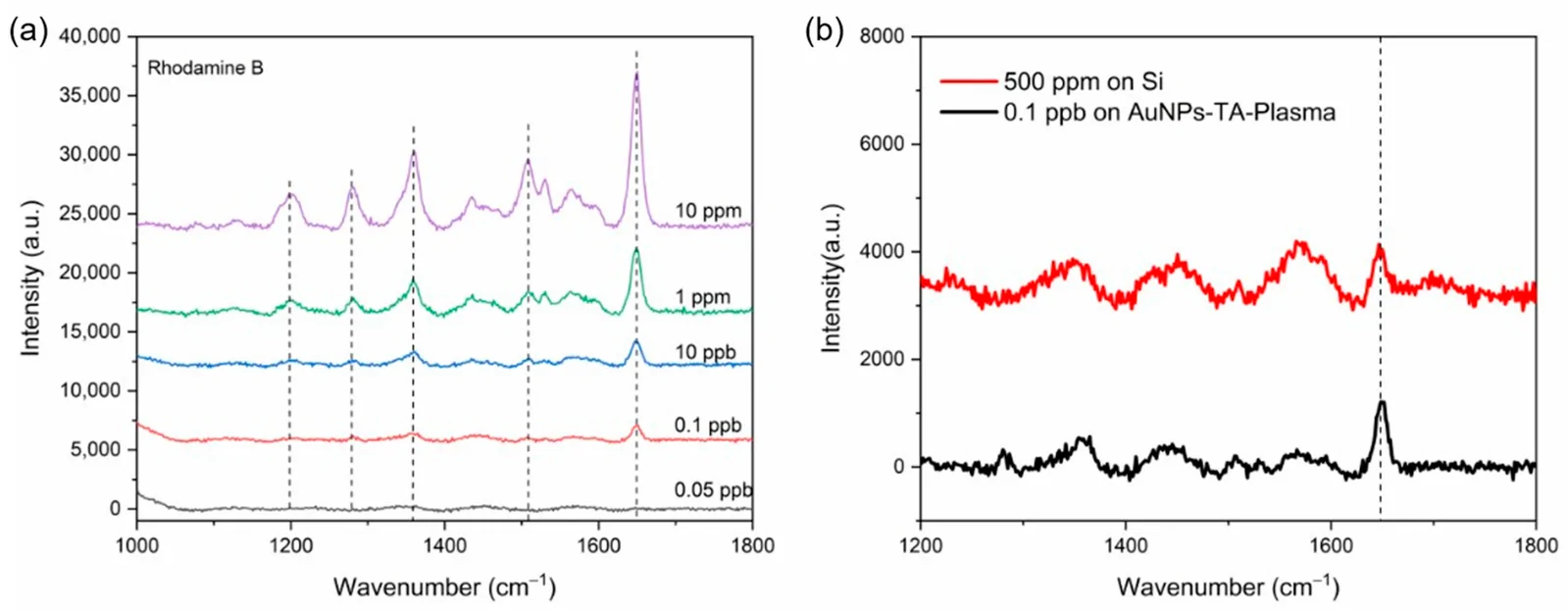
(**a**) SERS spectra of RhB at different concentrations on AuNPs-TA-Plasma substrates. (**b**) Raman spectra of 500 ppm RhB on Si substrate and SERS spectra of 0.1 ppb RhB on AuNPs-TA-Plasma.

We also tested the AuNPs-TA-Plasma substrate with rhodamine B (RhB), a common analyte widely used for SERS studies (Figure 18). The characteristic RhB bands appeared at 1198 cm^−1^ (aromatic C–H in-plane bending), 1280 cm^−1^ (C–C bridge stretching), and 1360, 1508, and 1650 cm^−1^ (aromatic C–C stretching of the xanthene ring) [40]. All main bands were resolved down to 1 ppb. The strongest band at 1650 cm^−1^ remained detectable at 0.1 ppb, whereas no RhB bands were distinguishable from the background at 0.05 ppb. The RhB detection limit is therefore about 0.1 ppb. This detection limit is comparable to several SERS systems reported previously [41,42]. We recorded a normal Raman reference spectrum of RhB on a bare Si wafer under the same acquisition conditions (Figure 18b) and calculated the SERS enhancement factor using the formula:

$$\mathrm{EF}=\frac{I_{\mathrm{SERS}}}{I_{\mathrm{Raman}}}\times \frac{C_{\mathrm{Raman}}}{C_{\mathrm{SERS}}}$$

Using the spectra obtained from 0.1 ppb RhB on SERS (AuNPs-TA-Plasma) and 500 ppm on Si, we obtained the EF of about 5.9 × 10^6^.

## 4. Conclusions

In this work, we combined an argon cold atmospheric-pressure plasma jet with a low dose of TA to synthesize AuNPs-TA-Plasma from HAuCl_4_ at room temperature, without additional harsh chemical reductants. The combination of TA and plasma treatment substantially improves AuNP synthesis. In situ UV–Vis spectroscopy showed that TA largely removed the induction period of plasma reduction. Under identical measurement conditions, AuNPs-TA-Plasma gave a stronger SERS response than AuNP samples prepared by conventional green synthesis with TA alone or by plasma treatment alone. This route offers a simple and energy-efficient way to prepare AuNPs. The particles have a surface accessible for sensing and catalysis, and the same strategy could be extended to other polyphenol-stabilized metal nanoparticles.

## Figures and Tables

**Table 1:** Comparison of the present plasma–TA synthesis with representative TA-based and plasma-based AuNP syntheses.

Stabilizer/Materials	HAuCl_4_ Concentration	TA:Au Molar Ratio	Size	SPR Peak	Volume	Time	Ref.
TA	0.2–2 mM	2:1–12:1	8–40 nm	—	—	Boiling 2 min + stirring 20–30 min	[37]
TA + trisodium citrate	2.5 × 10^−5^ mol	0.012:1–1.64:1	4.2–26.6 nm	—	100 mL	1 h + reflux 2 min	[36]
TA	0.13 mM	1.92:1	11.6 ± 3.2 nm	534 nm	50 mL	—	[38]
TA	NaAuCl_4_ 130 µM	0.18:1–18.46:1	27.6 ± 7.9–10.2 ± 1.7 nm	534 nm	52 mL	Boiling solution (7 min)	[39]
TA	0.2 mM	0.01:1	10.4 ± 2.4 nm	533 nm	10 mL/50 mL	10 min/30 min (Ar plasma jet)	This work

## Data Availability

The raw data supporting the conclusions of this article will be made available by the authors on request.

## Supplementary Materials

The following supporting information can be downloaded at: https://www.mdpi.com/article/10.3390/nano16211386/s1, Figure S1. UV–Vis spectra of plasma-treated water (PAW) and of PAW after the addition of HAuCl4 (initial, after 10 min and after 20 min). Figure S2. UV–Vis spectra of TA before and after plasma treatment (Plasma-TA) and of Plasma-TA after the addition of HAuCl4 (initial, after 10 min, and after 20 min). Figure S3. UV–Vis spectra of the AuNPs-TA-Plasma, AuNPs-TA, and AuNPs-Plasma colloids used for the SERS measurements.

## Abbreviations

The following abbreviations are used in this manuscript: AuNPs, gold nanoparticles; CAPPJ, cold atmospheric-pressure plasma jet; HRTEM, high-resolution transmission electron microscopy; SPR, localized surface plasmon resonance; MB, methylene blue; OES, optical emission spectroscopy; RhB, rhodamine B; SEM, scanning electron microscopy; SERS, surface-enhanced Raman scattering; TA, tannic acid; TEM, transmission electron microscopy; UV–Vis, ultraviolet–visible; XPS, X-ray photoelectron spectroscopy.
