# Supplementary material for: Rapid Plasma-Assisted Synthesis of Tannic Acid-Capped Gold Nanoparticles for SERS Applications
Source: Nanomaterials (Basel). 2026 Oct 28;16(21):1386. doi: 10.3390/nano16211386 (PMC13764805; doi:10.3390/nano16211386)
Supplement: Supplementary file 1 [file nanomaterials-16-01386-s001.zip › nanomaterials-4644718-supplementary.pdf]

Supplementary Information

Rapid Plasma-Assisted Synthesis of Tannic Acid-Capped Gold Nanoparticles for SERS Applications

Nguyen Thi Huyen[1,2,3], Nguyen Quynh Chi[4], Nguyen Thu Loan[4], Simon Chouteau[5], Jarl Tynan Collado[5], Pierre Vinchon[5], Vu Ngoc Linh[6], Man Minh Tan[1,2], Nguyen Hoang Tung[4,7], Nguyen Thanh Tung[4,7], and Nguyen Nhat Linh[4,7,*]

1 Laboratory of Advanced Materials and Natural Resources, Institute for Advanced Study in Technology, Ton Duc Thang University, Ho Chi Minh City, Vietnam

2 Faculty of Applied Sciences, Ton Duc Thang University, Ho Chi Minh City, Vietnam

3 Graduate University of Science and Technology, Vietnam Academy of Science and Technology, 18 Hoang Quoc Viet, Nghia Do, Hanoi, Vietnam

4 Institute of Materials Science, Vietnam Academy of Science and Technology, 18 Hoang Quoc Viet, Nghia Do, Hanoi, Vietnam

5 Osaka University, Suita, Osaka 565-0871, Japan

6 University of Engineering and Technology, Vietnam National University, VNU Hoa Lac Campus, Hoa Lac Commune, Hanoi 100000, Vietnam

7 University of Science and Technology of Hanoi, Vietnam Academy of Science and Technology, 18 Hoang Quoc Viet, Nghia Do, Hanoi, Vietnam

* Correspondence: linhnn@ims.vast.ac.vn

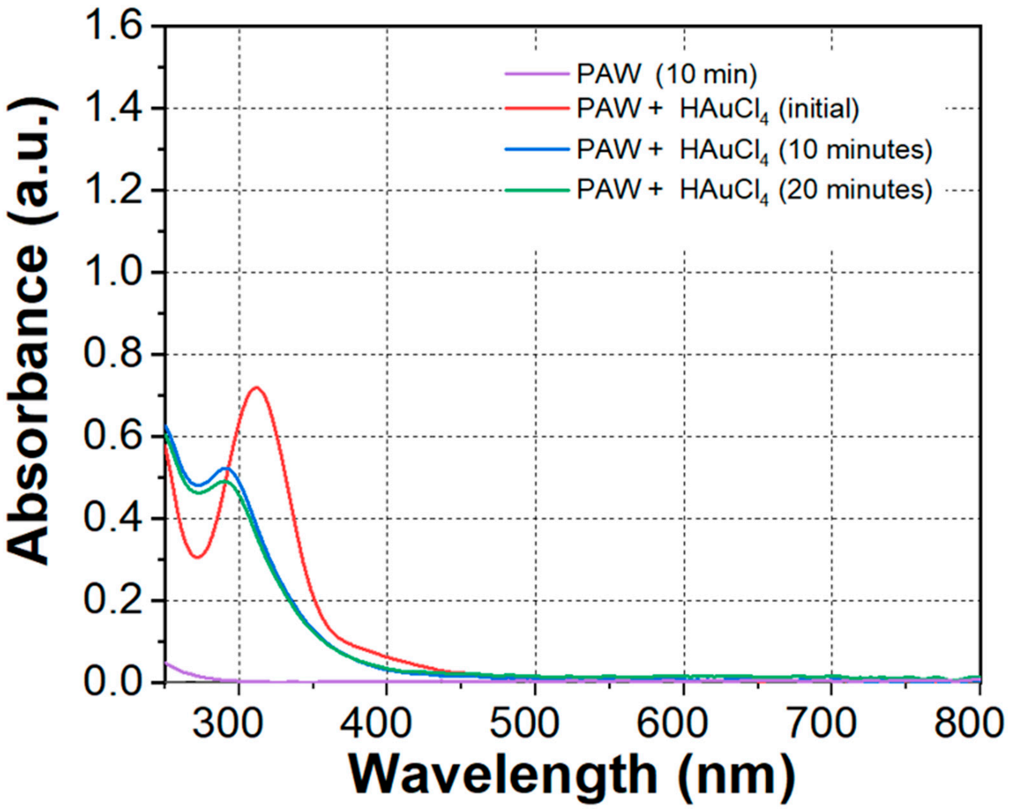

Figure S1. UV–Vis spectra of plasma-treated water (PAW) and of PAW after the addition of $HAuCl_4$ (initial, after 10 min and after 20 min).

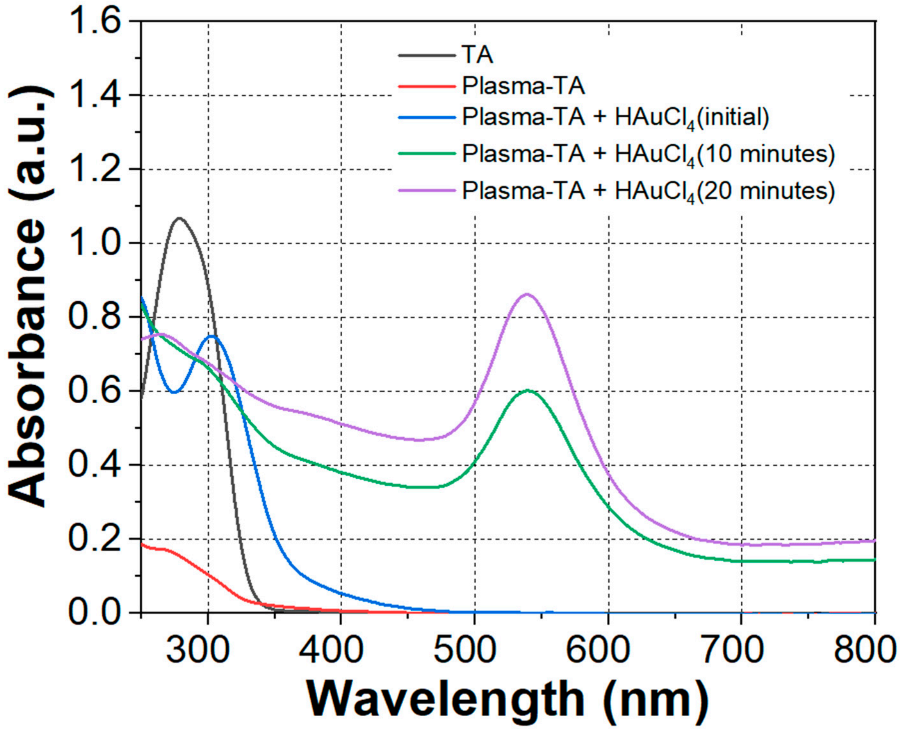

Figure S2. UV–Vis spectra of TA before and after plasma treatment (Plasma-TA) and of Plasma-TA after the addition of $HAuCl_4$ (initial, after 10 min, and after 20 min).

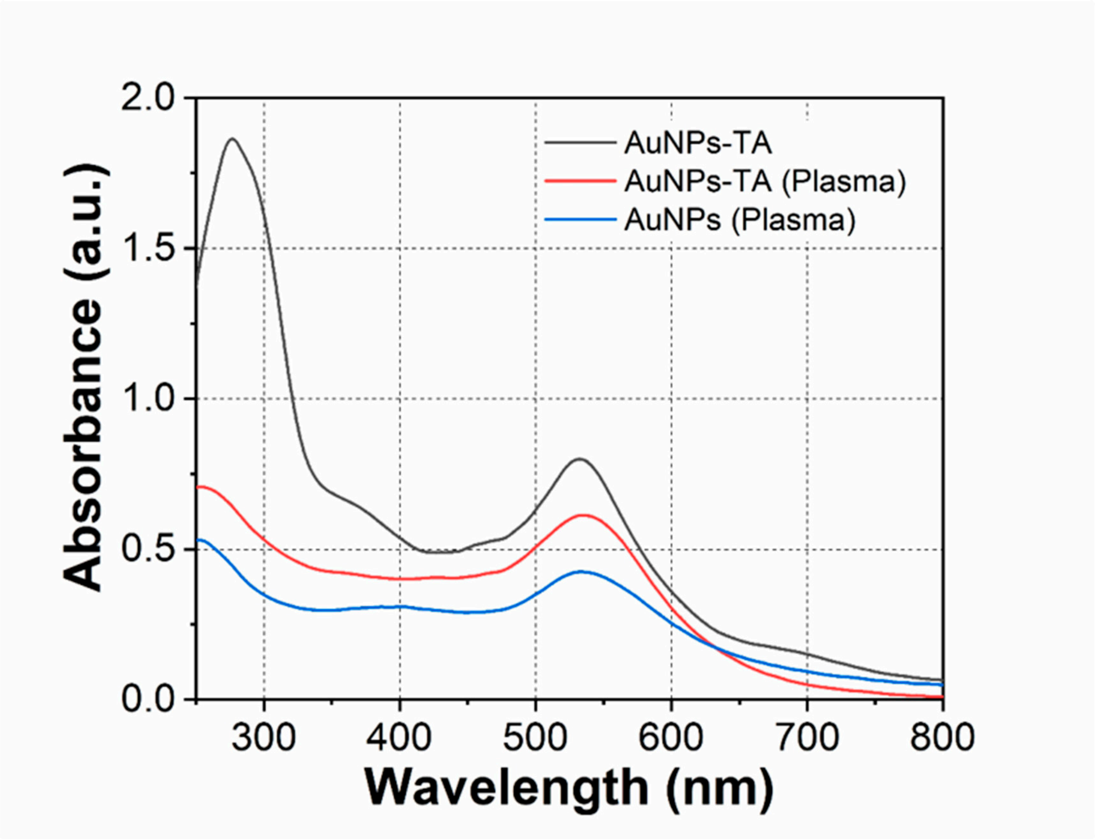

Figure S3. UV–Vis spectra of the AuNPs-TA-Plasma, AuNPs-TA, and AuNPs-Plasma colloids used for the SERS measurements.
